# The broad spectrum of unbearable suffering in end-of-life cancer studied in dutch primary care

**DOI:** 10.1186/1472-684X-11-12

**Published:** 2012-08-01

**Authors:** Cees DM Ruijs, Ad JFM Kerkhof, Gerrit van der Wal, Bregje D Onwuteaka-Philipsen

**Affiliations:** 1 Department of Public and Occupational Health, VU University Medical Centre, Amsterdam, The Netherlands; 2 Department of Clinical Psychology, VU University, Amsterdam, The Netherlands; 3 EMGO + Institute for Health and Care Research, Palliative Care Centre of Expertise, VU University Medical Centre, Amsterdam, The Netherlands

## Abstract

**Background:**

Unbearable suffering most frequently is reported in end-of-life cancer patients in primary care. However, research seldom addresses unbearable suffering. The aim of this study was to comprehensively investigate the various aspects of unbearable suffering in end-of-life cancer patients cared for in primary care.

**Methods:**

Forty four general practitioners recruited end-of-life cancer patients with an estimated life expectancy of half a year or shorter. The inclusion period was three years, follow-up lasted one additional year. Practices were monitored bimonthly to identify new cases. Unbearable aspects in five domains and overall unbearable suffering were quantitatively assessed (5-point scale) through patient interviews every two months with a comprehensive instrument. Scores of 4 (serious) or 5 (hardly can be worse) were defined unbearable. The last interviews before death were analyzed. Sources providing strength to bear suffering were identified through additional open-ended questions.

**Results:**

Seventy six out of 148 patients (51%) requested to participate consented; the attrition rate was 8%, while 8% were alive at the end of follow-up. Sixty four patients were followed up until death; in 60 patients interviews were complete. Overall unbearable suffering occurred in 28%. A mean of 18 unbearable aspects was present in patients with serious (score 4) overall unbearable suffering. Overall, half of the unbearable aspects involved the domain of traditional medical symptoms. The most frequent unbearable aspects were weakness, general discomfort, tiredness, pain, loss of appetite and not sleeping well (25%-57%). The other half of the unbearable aspects involved the domains of function, personhood, environment, and nature and prognosis of disease. The most frequent unbearable aspects were impaired activities, feeling dependent, help needed with housekeeping, not being able to do important things, trouble accepting the situation, being bedridden and loss of control (27%-55%). The combination of love and support was the most frequent source (67%) providing strength to bear suffering.

**Conclusions:**

Overall unbearable suffering occurred in one in every four end-of-life cancer patients. Half of the unbearable aspects involved medical symptoms, the other half concerned psychological, social and existential dimensions. Physicians need to comprehensively assess suffering and provide psychosocial interventions alongside physical symptom management.

## Background

In incurably ill patients preparing for the end-of-life concern occurs whether suffering will be unbearable [1,2]. For physicians, relief of suffering is an important goal of the care they provide [2,3]. Sometimes palliative interventions do not take away the suffering. An important question for physicians is to ask their patients whether the suffering is bearable. If unbearable suffering is present, physicians need to assess which aspects are unbearable, to adjust palliative interventions. In the contemporary debate about euthanasia and physician assisted suicide (EPAS) unbearable suffering is a frequently mentioned component [3-5]. Legislative criteria formulated in relation to EPAS require physicians to assess whether unbearable suffering is present [6]. Despite the relevance of the issue of unbearable suffering in medicine, little research addressing the nature of unbearable suffering is performed [7].

Unbearable suffering most frequently is reported in end-of-life cancer patients in primary care [6]. Tens of thousands end-of-life cancer patients on a yearly basis are cared for at home in primary care in various health care systems; the percentage of cancer patients dying at home varies between countries and states (13%-60%) [8-11]. Characteristics of palliative primary care include: palliative care delivered by a physician trained in general medicine, strong relationships between physicians and their patients, care provided at home and dependent upon the possibilities of care at home, support by a home-team, support by a specialized palliative care service [11-13], selection of patients with a preference to die at home [14,15] and negative selection of patients with cancer-related emergencies which require treatment in secondary care [9]. Systematic research investigating end-of-life cancer patients cared for in primary care is seldom performed.

Most of the research investigating end-of-life cancer patients is performed in secondary care and in hospices [16]. Frequent problems occur with the recruitment of a study population, despite the immediate availability of patients in these settings where cancer care is concentrated. Threats to recruitment include physician refusal, patient refusal and the difficulty to estimate life expectancy [17,18]. Recruitment of end-of-life cancer patients for research in primary care provides additional difficulties, because the patients are dispersed over many practices. Additional study organization is necessary in primary care to recruit a sufficiently large number of participating physicians (more participating physicians are necessary to realize an adequate patient study-population) , to identify the patients per practice and to arrange the provision of interviews at home.

In the Netherlands some 40.000 patients die from cancer each year, representing 28% of all annual deaths [8]. General practitioners (GPs) in 45% are the primarily responsible physicians for patients dying from cancer [8]. Primary care in the Netherlands nationwide is provided by some 8.000 GPs, 60% of whom work part time, together delivering care for the equivalent of 6.500 full-time practices [19]. A full time GP on average is responsible for palliative care for end-of-life cancer patients nearly three times a year.

We performed a cross-sectional study in cancer patients close to death in primary care and addressed the following questions: (1) which aspects of suffering are unbearable and what is the frequency; (2) what is the frequency of overall unbearable suffering, and; (3) what are sources of the capacity to bear suffering?

## Methods

### Design and population

The study was conducted in Utrecht, a city with a population of 235 000 people and 105 GPs. The inclusion criteria were (1) terminal cancer; (2) an estimated life expectancy of half a year or shorter; (3) mentally competent; (4) adequately fluent in Dutch; (5) expectedly living at home (most of the time) until death and; (6) having a GP as the primary responsible physician.

Complete case identification is recommended in palliative cancer care research [17]. Low physician recruitment rates (2%-49%) and over-restrictive gate-keeping [20-22] are reported in primary care research in relation to a failing partnership between researchers and GPs [23,24], indicating the risk of not indentifying all cases, or even a failing study. A methodological measure interfering with complete case identification is an a-priori exclusion of patients who are too gravely ill. Analysis of the recruitment barriers resulted in the conclusion that participation of committed GPs [23] and strict monitoring of newly eligible patients were essential requirements to realize complete case identification. Direct professional colleagues are without doubt committed participants, and therefore GPs were personally recruited throughout the city among professional colleagues of the first author (CDMR). A sufficiently large and dispersed GP sample was considered to serve a patient population representative of the city. Forty-four GPs participated, of whom 35 worked in eight group practices; 5 worked in three duo practices and 4 worked in solo practices. Eighteen of the GPs worked full time. The practice locations were dispersed throughout the city, with three fourths middle class and working class neighborhoods and one fourth deprived neighborhoods. Six GPs refused to participate. GPs identified eligible patients, informed them about the study, and requested whether a researcher would be permitted to explain the study. Consenting patients were visited within a week at their residence by a researcher, to explain the study and request definite participation. In participating patients demographic information was registered and the baseline interview administered, preferably in the first contact. Follow-up interviews were administered every two months at the patients’ residence or earlier, upon notice of the GP, if the general condition of a patient deteriorated.

A study coordinator was appointed to organize the recruitment process. To monitor the eligible population and stimulate recruitment, the study coordinator contacted the GPs every two months to request information on newly eligible patients. Characteristics of all eligible patients and reasons for not entering the interview study were registered. Patients were included from May 2003 until May 2006, follow-up continued until May 2007. Seventy six out of 148 invited patients (51%) entered the interview study. The main reason mentioned by patients (or family) for declining was a physical condition which unexpectedly deteriorated so rapidly that an interview was no longer possible (N = 27), followed by considering participation too burdensome (N = 20) and “don’t like talking” (N = 15). Another 110 advanced cancer patients were not invited by the GP, because unexpected rapid deterioration resulted in a too debilitated physical condition. The attrition rate in the interview sample was 8% caused by patients who stopped participating after one or more interviews. At the end of follow-up 8% of the patients were alive.

Age, sex and type of cancer did not differ between the interview sample and the sample who declined to participate. Depressed mood according to the GP was less prevalent in the interview sample than in the declining sample (5% versus 23%) [25]. The interviewers were the study coordinator (a physiotherapist) and a GP (CDMR); both were trained in interview techniques. The study protocol was approved by the Medical Ethics Committee at the VU University Medical Centre. Patients were clearly informed that they were free to decline from further participation at any time. The recruitment process is described in detail elsewhere [25].

### Definition

Unbearable suffering is a subjective experience in which the suffering is so serious and uncontrollable that it overwhelms one’s bearing capacity.

### Measurement instrument

Existing quality of life instruments were not suitable, because they frequently measure the intensity or extent of potentially suffering inducing aspects, but not whether suffering occurs. In the absence of a suitable measuring device, the State-of-Suffering-V (SOS-V) was developed: a quantitative instrument with a comprehensive design, measuring unbearable suffering in five domains. The development of the instrument, including analysis of validity, is described in detail elsewhere [26]. The instrument was required to include physical, psychological, social and existential dimensions of suffering [1,2,27-31]. Assessment of suffering requires a framework of suffering [32], which was required to include the domain of medical signs and symptoms, to assess the position of traditional medical aspects in the spectrum of suffering. A framework consisting of the following five domains provided a practical, functional construct: (I) medical signs and symptoms; (II) loss of function; (III) personal aspects; (IV) aspects of environment and (V) nature and prognosis of the disease.

Sixty nine suffering inducing aspects were identified in literature and attributed to the domains. Specific physical cancer induced aspects were largely present, since they relate to specific interventions. Other aspects permitted a more general formulation. The domain of environment included (lack of) support and aspects of social interaction, such as experiencing to be a burden to others and experiencing symptoms to be embarrassing, resulting in withdrawal from social interaction. Fear of future suffering, due to either progression of symptoms, or impairment of strength to bear the suffering, constitute two aspects which are frequently mentioned in Dutch literature concerning EPAS.

The scoring system paralleled the dynamics of suffering: first it is asked in which intensity (or extent) an aspect is present and then (if present) in how far the aspect is experienced to be unbearable. A uniform 5-point scoring scale is employed, with a description of scores: 1-not at all; 2-slightly; 3-moderately; 4-seriously; 5-very seriously, hardly can be worse. The subjective nature of suffering demands scores to be provided by the patients. After rating the specific aspects it is asked to name and rate any missing aspects of suffering. Thereafter the patient is asked to rate overall unbearable suffering (same scale). The interview ends with four open-ended questions investigating sources of bearing capacity and other relevant experience: (1) what gave you the strength to bear the suffering; (2) did faith or life conviction provide strength; (3) have you witnessed serious disease of relatives in the past and how did this affect the present suffering, and; (4) what, if any, were positive consequences of the disease? Exact phrases of the answers were immediately written down by the interviewer. The reference period was the last two days. Field testing was employed patients in a few patients before the start of inclusion, which demonstrated that the patients understood the questions, and that the instrument was easy to administer.

The SOS-V was administered together with other instruments, which included the Schedule for Clinical Assessment in Neuropsychiatry (SCAN) [33] at baseline, as part of a study which also investigated depression [34]. Administration of the quantitative questions of the SOS-V most times was possible within 15 to 20 minutes.

### Analysis

A cross-sectional analysis was employed using the results of the last administered SOS-V in each patient, indicating suffering close to death. Unbearability for aspects of suffering was analyzed dichotomously, employing a cut-off score. Aspect with scores 4 (seriously) and 5 (very seriously) were defined unbearable. Descriptive data are presented. Sources of bearing capacity were analyzed qualitatively. All literal formulations of sources were compiled in a table, corresponding answers were combined, codes were assigned and the sources per patient over all interviews were assessed. Only sources with a prevalence of 5% or more are presented.

## Results

### Interview sample

The studied interview sample consisted of 64 interviewed patients with follow up until death, of whom 46 died within 6 months after inclusion. In 4 patients no SOS-V could be administered, because of clinical deterioration. The last interview on average was 30 days before death (SD 17 days); in 23% the last interview was within 2 weeks prior to death; in 41 patients at least two interviews were administered.

The average age in the interview sample was 70 years (range 38–86), 52% were female, and the most prevalent cancer types were lung cancer (27%) and gastro-intestinal cancer (25%); 60% were educated beyond elementary school; 63% were living alone, 77% had children and 62% were religious (protestant or catholic). In the interview sample one patient suffered from a definite major depression [34].

### Unbearable suffering

**Figure 1:**
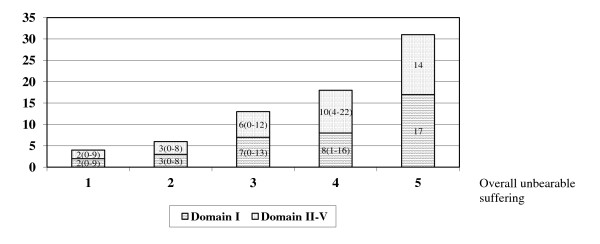
Average number and range of unbearable aspects in relation to overall unbearable suffering (n=57)*. *Three patients did not provide a score for overall unbearable suffering. Scale for overall unbearable suffering: 1:not present; 2:slightly; 3:moderately; 4:seriously; 5:very seriously, hardly can be worse.

Overall unbearable suffering occurred in 28% of the patients (26% serious; 2% very serious), with a mean number of 18 unbearable aspects for patients with serious unbearable suffering (score 4) and up to 31 unbearable aspects in one patient with very serious (score 5) overall unbearable suffering. Overall unbearable suffering was slightly present in 10% of the patients, moderately present in 25%, and was absent in 37%. Absent, slight and moderate overall unbearable suffering went along with respective means of 4, 6 and 13 unbearable aspects. The most frequent unbearable aspects (scores 4 or 5) divided over the domains of the SOS-V included: (I) *Medical signs and symptoms*: weakness, general discomfort, tiredness, pain, loss of appetite and not sleeping well (25%-57%) (Table 1), (II) *Loss of function*: impaired routine daily activities, impaired leisure activities, help needed with housekeeping and being bedridden (32%-55%)(Table 2), (III) *Personal aspects*: feeling dependent on others, not able to do important things, trouble accepting the situation and loss of control over one’s own life (27%-45%), (IV) *Environment*: relatives consider the suffering too severe (16%), practical loneliness (nobody present) (12%) and (V) *Nature and prognosis of disease*: fear of future suffering (17%). Two unbearable aspects were added: delayed income caused by insurance bureaucracy and the prospect of loss of beloved ones through death. The unbearable aspects were evenly divided over domain I and the collective domains II-V (Figure 1).

**Table 1:** Unbearable aspects in the domain “Medical signs and symptoms” (n=60)

	**Unbearable, score 4 or 5**^**a**^	**Aspect present**
	**% (n)**	**% (n)**
Aspect of suffering		
Domain 1: Medical signs and symptoms		
Weakness	57 (34)	93 (56)
General discomfort	37 (22)	80 (48)
Tiredness	35 (21)	87 (52)
Pain	25 (15)	72 (43)
Loss of appetite	25 (15)	62 (37)
Not sleeping well	25 (15)	47 (28)
Changed appearance	22 (13)	78 (47)
Vomiting	20 (12)	27 (16)
Shortness of breath	19 (11)	59 (35)
Impaired co-ordination	18 (11)	57 (34)
Loss of concentration	17 (10)	40 (24)
Incomprehensible speech	15 (9)	32 (19)
Memory loss	15 (9)	43 (26)
Nausea	13 (8)	28 (17)
Smelling unpleasant	13 (8)	35 (21)
Impaired hearing	13 (8)	33 (20)
Swallow food impaired	12 (7)	36 (21)
Feeling depressed	12 (7)	34 (20)
Feeling tense	12 (7)	44 (26)
Dizziness	12 (7)	27 (16)
Constipation	12 (7)	30 (18)
Impaired mental clarity	12 (7)	42 (25)
Thirst	12 (7)	45 (27)
Hiccups	10 (6)	22 (13)
Intestinal cramps	8 (5)	22 (13)
Itch	7 (4)	32 (19)
Impaired sight	7 (4)	42 (25)
Swallow fluid impaired	7 (4)	23 (14)
Diarrhea	7 (4)	20 (12)
Feeling anxious	7 (4)	27 (16)
Incontinence of feces	7 (4)	8 (5)
Coughing	5 (3)	38 (23)
Pressure ulcers	3 (2)	8 (5)
Impaired comprehension of speech	3 (2)	7 (4)
Skin metastasis	2 (1)	3 (2)
Paralyzed limbs	2 (1)	5 (3)
Incontinence of urine	0 (0)	10 (6)
Rounded percentages and absolute numbers; between 0 and 1 missing observations per aspect		

^a^Unbearability was measured on a 5-point scale: 1:not present; 2:slightly; 3:moderately; 4:seriously; 5:very seriously, hardly can be worse.

**Table 2:** Unbearable aspects in the domains “Loss of function”, “Personal aspects”, “Environment” and “Nature and prognosis of disease” (n=60)

	**Unbearable, score 4 or 5**^**b**^	**Aspect present**
	**% (n)**	**% (n)**
Aspect of suffering		
Domain 2: Loss of function		
Impaired routine daily activities	55 (33)	83 (50)
Impaired leisure activities	50 (30)	82 (49)
Help needed with housekeeping	42 (25)	71 (42)
Bedridden	32 (19)	56 (33)
Help needed with self-care	22 (13)	60 (36)
Impaired working capacity^c^	12 (7)	17 (10)
Impaired sexuality	5 (3)	14 (8)
Domain 3: Personal aspects		
Feeling dependent on others	45 (27)	80 (48)
Not able to do things you consider important	42 (24)	63 (36)
Trouble accepting the present situation	33 (20)	60 (36)
Loss of control over your own life	27 (16)	30 (18)
Negative thoughts or worrying	15 (9)	32 (19)
Hopelessness	13 (8)	28 (17)
Feeling a nuisance to others	13 (8)	38 (23)
Feeling lonely (intrapersonal)	10 (6)	20 (12)
Feeling not any longer being the same person	10 (6)	28 (17)
Feelings of worthlessness	10 (6)	22 (13)
Feeling tired of life	9 (5)	17 (10)
Feeling of no longer being important to others	8 (5)	18 (11)
Experienced little happiness with family/friends	8 (5)	22 (13)
Not satisfied with your own self	7 (4)	12 (7)
Feelings of guilt	5 (3)	12 (7)
Lived a life with little purpose	3 (2)	8 (5)
Experienced little success in life	2 (1)	10 (6)
Domain 4: Environment		
Relatives consider your suffering too severe	16 (9)	33 (19)
Practical loneliness (no one present for you)	12 (7)	15 (9)
Insufficient availability of care	8 (5)	12 (7)
Unsatisfactory social contacts	3 (2)	8 (5)
Insufficient support (family, friends, those nearby)	2 (1)	5 (3)
Shame (socially embarrassing symptoms)	2 (1)	2 (1)
Domain 5: Nature and prognosis of disease		
Fear of future suffering	17 (10)	40 (24)
Fear of future failing strength to bear suffering	10 (6)	25 (15)
Rounded percentages and absolute numbers; between 0 and 3 missing observations per aspect		

^b^Unbearability was measured on a 5-point scale: 1:not present; 2:slightly; 3:moderately; 4:seriously; 5:very seriously, hardly can be worse.

^c^50 persons indicated they did not work. Of 10 working persons 70% rated the impaired working capacity unbearable.

### Sources of bearing capacity and other relevant experience

The most frequent sources providing strength to bear the suffering were “love and support (generally mentioned together) by family and relatives” (67%), and “faith and trust in God” (40%). Other frequent sources were: “optimism and positivity” (27%), “acceptance” (18%), “maintenance of daily routine” (18%) and “living by the day” (13%) (Table 3).

**Table 3:** Bearing capacity: what gives you the strength to bear the suffering ?* (n=60)

	%
-Love and support from family and relatives	67
-Faith in God, prayer**	40
-Positivity, optimism, enjoy what is possible	27
-Acceptance	18
-Concentrate on maintaining ones daily routine, keep on living	18
-Live by the day, don’t think about what may happen in the future	13
-Inner strength	10
-Humor	7
-Accomplish tasks	7
-Confidence, no fear of the future	5
-Fighting spirit	5
-Shut of feelings of sorrow or loss	5
-Hope to remain some more time	5

More than one answer possible.

Number of interviews: 152 (median: 2 per patient; range:1-11).

*only answers mentioned in 5% or more.

**direct question about the influence of faith.

Eighty percent of the interviewed patients had previously witnessed relatives seriously suffering from disease; in 13% of the interview sample the experience resulted in increased present suffering (caused by increased fear of future suffering), while in 12% the result was increased confidence to bear future suffering (caused by witnessing a person who managed to deal positively with suffering). Thirty-eight percent of the patients experienced positive consequences of some kind in relation to their disease, such as growth of contact with beloved ones (52%), growth as a person (26%) and re-establishing contact with beloved ones (in present life) (17%) (data not in table).

## Discussion

Overall unbearable suffering occurred in one in every four of end-of-life cancer patients. Overall, half of the unbearable aspects occurred in the domain of medical signs and symptoms. The most frequent unbearable aspects were weakness, general discomfort, tiredness, pain, loss of appetite and not sleeping well (25%-57%). The other half of the unbearable aspects occurred in the collective domains of loss of function, personal aspects, environment and nature and prognosis of disease. The most frequent unbearable aspects were impaired capacity to perform activities, feeling dependent, help needed with housekeeping, not being able to do important things, trouble accepting the situation, being bedridden and loss of control (27%-55%). Important sources providing strength to bear the suffering were love and support by family and relatives, faith in God, positivity, acceptance, maintain daily routine and living by the day. Previous witnessing of serious suffering caused by disease sometimes resulted in anticipatory fear, yet others gained confidence to sustain suffering. Frequently patients also experienced some positive influence caused by their disease.

The recruitment proportion of patients requested to participate in our study was 51% , which corresponds to other studies such as those of Kuupelomäki [35](46%), Steinhauser [36](46%), Tishelman [37](47%) and Wilson [27](50%) investigating characteristics of end-of-life cancer patients, but was lower than Balboni [38](63%) and Chochinov [39](72%), taking into account some differences in composition of the data. All of these studies, with the exception of the study of Steinhauser (database recruitment strategy), were in easier to approach secondary care patient populations. Further, our study monitored all end-of-life cancer with a life expectancy of half a year or shorter and identified 110 patients who were too gravely ill to enter the protocol, demonstrating yet again the difficulty to predict life expectancy. In several studies investigating end-of-life cancer patients a physical condition not permitting an interview frequently was an a priori exclusion criterion [27,35,38,39], which in end-of-life studies interferes with knowing the total population of dying cancer patients present in the studied setting; we therefore could not compare for this aspect.

Limitations of our study include the small sample size and the small number of interviews shortly before death; both may be related to the difficulty to predict life expectancy in end-of-life cancer patients [17]. The use of a largely untested instrument is another limitation. Furthermore, the proportion of patients with depressed mood according to the GP was lower in the interview sample. In how far this is representative for prevalence of major depression is difficult to say, because the presence of major depression frequently cannot be predicted based on primary care consultations [40]. In the other studies investigating end-of-life cancer patients [27,35-39,41] presence of depressed mood in the out-of-study population was not analyzed and comparison cannot be made. The outcomes of our study relate to an urban primary care population and cannot be generalized. Further, with respect to primary care, differences between health care systems [8] need to be accounted for. Still, core aspects of suffering are universal [1,2,42].

Various studies have addressed “suffering”. Wilson et al. [27] studied patient-rated overall suffering in relation to 21 interviewer-rated symptoms and concerns in 381 secondary care advanced cancer patients; moderate to extreme overall suffering occurred in 26% of the patients. Benedict [41] studied overall suffering and 26 aspects of suffering in 30 secondary care lung cancer patients with and without metastasis; very much suffering was reported by 50% of the sample. Kuuppelomäki et al. [35] studied overall suffering rated by 32 secondary care patients with incurable cancer; moderate to very intensive overall suffering was present in 81% of the patients. Aspects of suffering which emerged in the various studies [27,35,41] included: general malaise, weakness, pain, fatigue, depression, anxiety, changed daily activities, negative body changes, dependence, helplessness and restricted social life. Pasman et al. [7], in a qualitative investigation of unbearable suffering in chronically ill patients (mainly non-cancer) found an emphasis of patients on psychosocial suffering. Comparison of the results of these studies with our study is difficult, due to differences in study design, setting, patient characteristics, and employed cut-off scores. Yet, serious overall suffering in end-of-life cancer patients frequently occurred in all settings. Our study addressed unbearable suffering; in how far the suffering was unbearable was not investigated in the other studies.

The outcomes of studies investigating suffering of patients need to be assessed in relation to the providers of care. Studies investigating the assessment of unbearable suffering by physicians [7,43] indicated a focus on assessing physical suffering. Also in consultations provided to physicians (many being GPs) by palliative care consultation teams physical problems were the discussed topic in 77% of consultations [12], while aspects of spiritual care played a role in only 8% of consultations [44]. These findings contrast with our study, which demonstrated half of the unbearable aspects of suffering to be psychological, social or existential in nature. It may be that psychological, social and existential aspects of suffering are underrecognized. Another possibility may be that physicians consider these aspects to be part of the personal domain of patients.

Palliative care is total care and requires understanding of the full diversity of the contributing aspects [45-47]. The taxonomy of unbearable suffering employed in our study helps to maintain an overall structured view on aspects contributing to suffering, even if the division of suffering into physical and non-physical determinants [1,47], and likewise the division of the human condition into medical and non-medical domains, is arbitrary [2]. In our study half of the unbearable aspects of suffering occurred in the traditional medical domain of signs and symptoms. The other half occurred in the psychological domain in a broader sense, comprising psychological, social and existential aspects of suffering [1,27,48]. The suffering in the domains II-V of the measuring instrument indicates various aspects of loss, such as loss of autonomy (loss of control, feeling dependent, help needed), loss of personal role and perspective of the future (impaired activities, not able to do important things), loss of social functioning (feel a nuisance, no longer feel important to others), loss of existential well-being (feel worthless, feel tired of life), loss of appreciation of self (no longer feel to be the same person, not satisfied with own self) and loss of certainty caused by the unpredictable course of the disease (fear of future suffering). Through these losses persons become disconnected from their ordinary world [2] and the meaning of their lives [1,42].

The importance of psychosocial, psychotherapeutic and spiritual interventions to relieve suffering and restore meaning in advanced cancer patients is acknowledged and individual and group psychotherapy for advanced cancer patients has been developed [30,32,49-53], with recent studies focusing on meaning-centered interventions [30,49,50] and dignity [51]. However, not much is known about the effects of psychosocial and spiritual palliative interventions to relieve suffering in patients who are residing at home and are close to death. An important step to provide such interventions is to assess the nature of psychological (psychological, social, existential) suffering and understand it’s meaning for the patient, as well as to assess the physical aspects of suffering, taking into account that one may affect the other. Also, the sources providing strength to bear suffering should be assessed, because they indicate opportunities to relieve suffering [2,46].

## Conclusions

One in every four end-of-life cancer patients in this study in primary care overall suffered unbearably. Half of the unbearable aspects occurred in the psychological domain in a broad sense, indicating the need for training physicians in skills of explicitly assessing psychological, social and existential suffering and in the provision of psychosocial and spiritual interventions, alongside skills for medical diagnostics and providing medical interventions directed at diminishing the intensity of physical aspects. Studies investigating unbearable suffering closer to death are required.

## Competing interests

The authors declare that they have no competing interests.

## Authors’ contributions

CDMR had the initial idea for this study and wrote the initial research proposal. AJFMK , GVDW and BDO-P commented on and contributed to the final research proposal. CDMR and BDO-P did the analysis, which was discussed with AJFMK and GVDW. CDMR wrote the first draft. AJFMK, GVDW and BDO-P commented on and contributed to the final draft. All contributors had access to all the data and can take responsibility for the integrity of the data and the accuracy of the data analysis. All authors read and approved the final manuscript.

## Ethical approval

The study protocol was approved by the Medical Ethics Committee at the VU University Medical Centre (METC VUmc No. 2002/79).

## Fundings

This study was supported by the Netherlands Organization for Scientific Research (NWO) (015.01.080); the Aspasia Program. The funding organization was not involved in the conduct of the study.

## Pre-publication history

The pre-publication history for this paper can be accessed here:

http://www.biomedcentral.com/1472-684X/11/12/prepub
